# Influence of Aging on the Flexural Strength of PLA and PLA-X 3D-Printed Materials

**DOI:** 10.3390/mi15030395

**Published:** 2024-03-14

**Authors:** Nenad Mitrović, Zorana Golubović, Aleksandra Mitrović, Milan Travica, Isaak Trajković, Miloš Milošević, Aleksandar Petrović

**Affiliations:** 1 University of Belgrade, Faculty of Mechanical Engineering, 11000 Belgrade, Serbia; zzgolubovic@mas.bg.ac.rs (Z.G.); apetrovic@mas.bg.ac.rs (A.P.); 2 The Academy of Applied Technical Studies, 11000 Belgrade, Serbia; amitrovic@atssb.edu.rs; 3 Innovation Center of Faculty of Mechanical Engineering, 11000 Belgrade, Serbia; mtravica@mas.bg.ac.rs (M.T.); itrajkovic@mas.bg.ac.rs (I.T.); mmilosevic@mas.bg.ac.rs (M.M.)

**Keywords:** 2D digital image correlation method, three-point bending, biomaterial testing, 3D printing, PLA, PLA-X, biomedical application

## Abstract

The three-point bending test is a valuable method for evaluating the mechanical properties of 3D-printed biomaterials, which can be used in various applications. The use of 3D printing in specimen preparation enables precise control over material composition and microstructure, facilitating the investigation of different printing parameters and advanced materials. The traditional approach to analyzing the mechanical properties of a material using a three-point bending test has the disadvantage that it provides only global information about the material’s behavior. This means that it does not provide detailed insight into the local strain distribution within the material. However, the 2D Digital Image Correlation (DIC) method offers additional insight, especially in terms of strain localization. DIC is an optical technique that measures full-field displacements and strains on the surface of a sample. PLA and enhanced PLA-X material were utilized to create three-point bending samples. The aim of this paper was to analyze and compare the influence of aging on the mechanical properties of PLA and enhanced PLA-X materials using three-point bending coupled with the DIC method. The results showed statistically significant differences between the PLA and PLA-X, for both the new and aged materials. The aged PLA samples had the highest average value of maximal force around 68 N, which was an increase of 8.8% compared to the new PLA samples. On the other hand, the aged PLA-X material had an increase of 7.7% in the average maximal force compared to the new PLA-X samples. When comparing the two materials, the PLA samples had higher maximal force values, 6.2% for the new samples, and 7.3% for the aged samples. The DIC results showed that both the new PLA and PLA-X samples endured higher strain values at Points 1 and 2 than the aged ones, except for the aged PLA-X sample at Point 2, where the new sample had higher strain values. However, for the first 5 min of the experiment, both materials exhibited identical behavior, after which point significant differences started to occur for both materials, as well as at Points 1 and 2. A more profound comprehension of the biomechanical characteristics of both PLA and PLA-X material is essential to enhance the knowledge for potential biomedical applications. The DIC method was found to be a powerful tool for analyzing the deformation and failure behavior of samples and for complementing the traditional approach to material testing.

## 1. Introduction

The three-point bending test is a widely used method for evaluating the mechanical properties of materials in both engineering and biomedical applications, including those manufactured using 3D printing technology. It is a standard method used to assess the mechanical behavior of materials under bending loads, involving the application of the load to a sample at one point and support at two points, creating a bending moment. This test provides valuable information on the strength of the material [1,2]. The applicability of the three-point bending test extends beyond traditional materials, such as metals and ceramics, to include flexible electronics and microfluidic devices. For example, researchers have used bending setups to investigate the reliability of flexible electronics subjected to repeated bending [3]. In the field of microfluidics, the three-point bending test has been employed to evaluate the mechanical properties of 3D-printed micro-fluidic devices, which are used in chemical and biomedical applications [4]. These devices often require good sealing and bonding between different materials, and the three-point bending test can assess their structural integrity and performance [5].

In the case of 3D-printed specimens, the three-point bending test can be used to evaluate their structural integrity, durability, and performance under bending loads, as well as their suitability for specific applications. Three-dimensional printing, also known as additive manufacturing, is a process that builds three-dimensional objects layer by layer from a digital model. It has gained significant attention in recent years due to its ability to produce complex geometries and customized designs, making it suitable for a wide range of applications, including biomedical applications [4,6]. One of the key advantages of using 3D printing in sample preparation for the three-point bending test is the ability to precisely control the printing parameters. This level of control enables researchers to investigate the effects of different printing parameters, such as layer height, filament width, and infill pattern, on the mechanical properties of the specimens [7]. Furthermore, 3D printing allows the application of advanced materials, such as nanoporous alumina films or polylactic acid (PLA), which can exhibit unique fracture behavior and mechanical properties [2,7]. However, the mechanical properties of 3D-printed materials can vary depending on various factors such as printing parameters, material composition, and infill density [8]. Recent years have been devoted to the challenging development of various biomaterials with specific properties that can be used in medicine and tissue engineering [9]. PLA as a thermoplastic polymer has shown versatile mechanical properties, biocompatibility, and biodegradability. Among other polyesters, such as PCL and PGA, PLA is used for the production of various covering membranes, drug delivery systems and devices [10], bioabsorbable medical implants of various types, scaffolds for tissue engineering [11,12,13,14], dental restorations, etc. [15,16,17].

The mechanical properties of 3D-printed materials can change over time due to various factors, including the ageing effect. Ageing refers to changes that occur in the material’s properties over time as a result of exposure to environmental conditions or internal chemical reactions. In the case of 3D-printed materials, the ageing effect can be influenced by factors such as heat treatment, hydrolytic degradation, and the process parameters used during printing. The research conducted by Hasan et al. [18] investigated the impact of ageing and heat treatment on the tensile properties of PLA printed parts. The study found that the ageing effect, combined with heat treatment, resulted in changes in the tensile properties of printed parts. Mechanical properties, such as tensile strength and elongation at break, were found to decrease over time, indicating degradation of the material. Ebrahimi and Ramezani Dana have shown that high temperature and humidity can accelerate PLA degradation within weeks to months [11]. Another study by Andrzejewska [19] focused on evaluating changes in material properties in PLA parts under various hydrolytic degradation conditions. The research examined the mechanical behavior of PLA specimens over a one-year period. The study found that the degradation medium had a significant influence on changes in mechanical behavior, including tensile strength and elongation at break. The results highlighted the importance of considering the effects of ageing and degradation conditions on the mechanical properties of 3D-printed materials. Furthermore, the process parameters used during printing can also affect the mechanical properties and ageing behavior of 3D-printed materials. Chacón et al. [20] investigated the effect of process parameters on the mechanical properties of PLA structures fabricated using fused deposition modelling (FDM). The study found that the selection of process parameters, such as layer thickness and raster angle, had a significant impact on the mechanical properties of printed parts. A study by Amza et al. [21] investigated the impact of accelerated aging, specifically UV-B exposure, on the mechanical properties of common 3D-printed polymers, with a focus on PLA. The research found that the stiffness of the 3D-printed parts did not change significantly after UV-B exposure. However, it was noted that creep behavior was closely related to decreased mechanical properties. This suggests that while the stiffness remained relatively stable, there were observable changes in the creep behavior and overall mechanical properties of the 3D-printed PLA parts after exposure to UV-B radiation. The study by Kalinke et al. [22] provided valuable insights into the influence of filament aging and conductive additives, particularly graphene, on the performance of 3D-printed sensors, which is highly relevant for understanding the potential impact of aging on the flexural strength of 3D-printed materials. The researchers emphasized the importance of optimizing the process parameters to achieve the desired mechanical properties and minimize the effect of aging.

PLA, derived from bioresources, is a biodegradable nontoxic organic polymer. This material is dimensionally stable and easy to extrude at low temperatures, so the platform does not need to be heated. It melts at low temperatures of 180–220 °C and has a glass transition temperature of 60–65 °C [23,24]. PLA exhibits certain challenging characteristics, notably in terms of thermal resistance, and these properties also have an impact on its mechanical behavior. PLA is known to have limitations in terms of thermal resistance, implying that it may not withstand high temperatures well. This characteristic can be of concern in applications where exposure to elevated temperatures is expected. The performance and structural integrity of PLA may be compromised under certain temperature conditions, leading to potential challenges in its mechanical behavior. Some properties of PLA can be improved by adding polymer mixtures, composites, or second-phase particles into the polymer matrix. The addition of other particles and polymers to the base material, in this case PLA, influences the possibility of changing the degradation rate and the mechanical and thermal properties of the material [25]. In terms of the fracture behavior of the modified material, referred to as PLA-X, the addition of second-phase particles to the PLA base material results in a significant enhancement in both the overall strain and the calculated tensile toughness. The PLA-X utilized in this study is an enhanced variant of PLA, characterized by the incorporation of second-phase particles with a melting temperature in the range of 190–220 °C [24,26]. This indicates that the inclusion of these particles has a positive effect on the material’s ability to withstand deformation and absorb energy before fracture occurs [22].

To enhance the understanding of the mechanical properties of 3D-printed specimens during the three-point bending test, additional tools such as Digital Image Correlation (DIC) can be employed. DIC is an optical technique that measures full-field displacements and strains on the surface of a sample by analyzing images captured during the test [27,28]. It is a useful method that promises significant applications in biomedical research, biomaterials, and tissue engineering. There is a demand for proper surface preparation for testing, after which DIC can be applied to engineering and biological materials [29]. The research of biomaterials and their applications is challenging. It is based on measurements that can lead to novel findings in applied research, quality assurance, and technology development. One of the challenges is the size and sensitivity of the samples, making strain measurement difficult. Using DIC, researchers can obtain detailed information on the strain distribution, strain localization, and deformation behavior of the specimen during the bending test, which can provide information on its mechanical properties and failure mechanisms [30,31,32,33]. 

The aim of this paper was to analyze and compare the influence of aging on the mechanical properties of 3D-printed samples made of PLA and enhanced PLA-X materials for biomedical applications using the three-point bending test coupled with the 2D Digital Image Correlation method.

## 2. Materials and Methods

The study used two commercial thermoplastic polymers: PLA (RepRap, Feldkirchen, Germany) and improved polylactic acid (PLA-X, Mitsubishi Chemical, Japan). The diameter of the filament of both materials was 1.75 mm.

**Figure 1:**
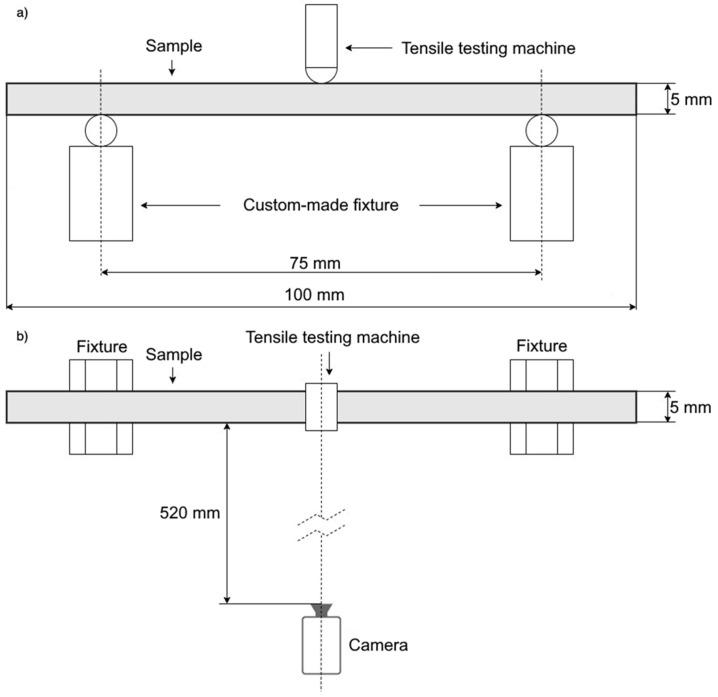
Graphic representation of the modeled samples: (**a**) Scheme of the three-point bending setup. (**b**) Scheme of the 2D DIC setup.

The three-point bending sample used in this study was designed in SolidWorks CAD 2023 software, with dimensions of 100 × 5 × 5 mm, Figure 1. 

**Figure 2:**
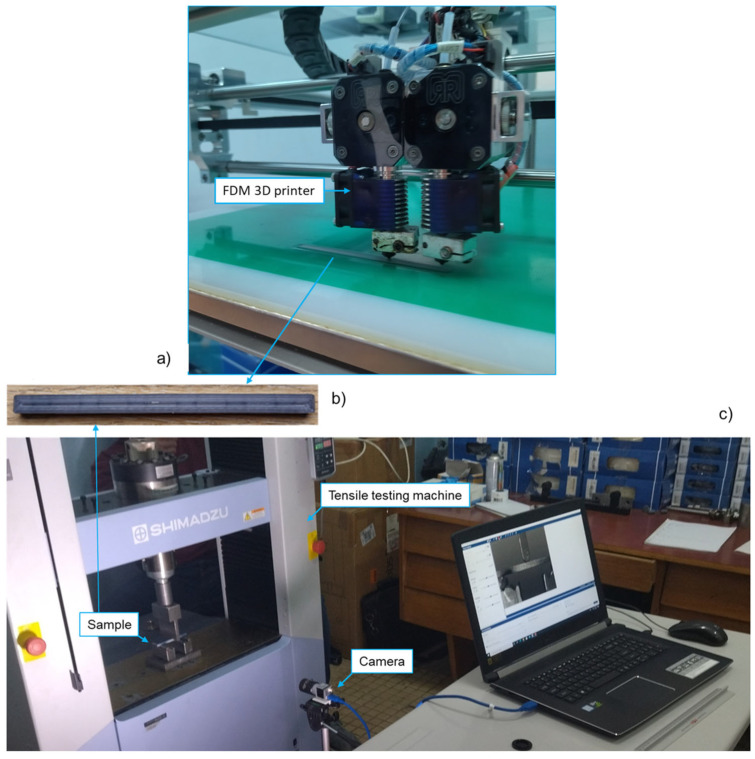
Experimental setup: (**a**) FDM 3D printing device; (**b**) printed sample; (**c**) DIC experimental setup.

The samples were printed using the fused deposition modelling (FDM) RepRap X400 3-D printer (Rep Rap, Feldkirchen, Germany). The infill pattern for all the samples was concentric with 30% infill density. The layer height was 0.2 mm, with 2 layers on the top, 2 on the bottom, and 2 from both sides. The nozzle temperature was set to an average of 195.5 °C, the nozzle diameter to 0.4 mm, and the printing speed was 60 mm/s. The temperature of the printing chamber was 33.2 °C, and the temperature of the platform was 48.1 °C. Three samples were printed for each material (PLA and PLA-X) and aged for two weeks in an open plastic box without exposure to light, at a constant room temperature of 23 °C and a humidity of 55% RH. After two weeks, a new set of samples was printed for each material. All samples were tested after printing the new set of samples. Four groups of samples with 12 samples in total were prepared. The concentric infill pattern was used for all samples with 30% of the infill density. The layer height was 0.2 mm, with 2 layers on the top, bottom, and on both sides (Figure 2b). 

All experiments were performed on the Shimadzu AGS-X tensile testing machine (Shimadzu, Japan), with a test speed of 1 mm/min. A custom-made fixture for three-point bending was designed, with a support span of 75 mm, as shown in Figure 1. For the application of 2D-DIC, a black-and-white stochastic pattern was applied on the sample surface to clearly allocate the pixels in the camera images (facets). Consequently, the pixel area of the reference image could be assigned to the corresponding pixel area of the target image. All images were recorded using a 35 mm lens and an acA-2500 Basler camera (Basler, Germany) with 5 MP camera resolutions. The camera was positioned 520 mm from the sample (Figure 2c). GOM Correlate (GOM, Germany) was used for the 2D-DIC application. Continuous specimen lighting was provided by an LED lamp.

Statistical analysis of flexural stress was performed in Excel 365 (Microsoft, Redmond, WA, USA). Data were analyzed using a standard *t*-test at the level of significance set at 0.05. A comparison of flexural stress was performed for four scenarios: aged and new PLA samples, aged and new PLA-X samples, new PLA and PLA-X samples, and aged PLA and PLA-X samples.

## 3. Results and Discussion

The mechanical properties of 3D-printed materials can change over time as a result of the ageing effect. Factors such as heat treatment, hydrolytic degradation, and process parameters during printing can influence the aging behavior and subsequent changes in mechanical properties. Understanding and considering these factors is crucial to ensure the performance and reliability of 3D-printed components.

The influence of the highest value of maximal force in three-point bending of 3D-printed specimens is a critical aspect in materials science and engineering. The highest value of maximal force plays a critical role in assessing material strength, ensuring quality control, predicting performance, guiding optimization efforts, and facilitating comparative studies within the additive manufacturing field. The maximal force represents the peak load that the material can withstand before failure. Therefore, it serves as a key indicator of the material’s strength under bending loads. A higher maximal force suggests greater resistance to deformation and fracture, indicating a stronger material. The maximal force recorded on the tensile testing machine for every sample, as well as the average force value and standard deviation for each sample group, are presented in Table 1. The flexural stress value was calculated for each sample, as well as the average force value for each sample group. The standard deviation values were low, which meant that all the measured values within a sample group were consistent (Table 1). The aged PLA samples had the highest average value of maximal force around 68 N, which was an increase of 8.8% compared to the new PLA samples. On the other hand, the aged PLA-X material had an increase of 7.7% in the average maximal force compared to the new PLA-X samples. When comparing the two materials, the PLA samples had higher maximal force values, 6.2% for the new samples, and 7.3% for the aged samples. Monitoring the highest maximal force is crucial for quality and integrity control in additive manufacturing processes. It helps ensure that the printed specimens meet the desired mechanical properties and structural integrity requirements. Deviations from expected maximal force values may indicate inconsistencies in printing parameters, material properties, or processing conditions. Low standard deviation values (Table 1) in the sample groups indicated that the sample within each group exhibited similar mechanical properties and response to bending loads.

**Figure 3:**
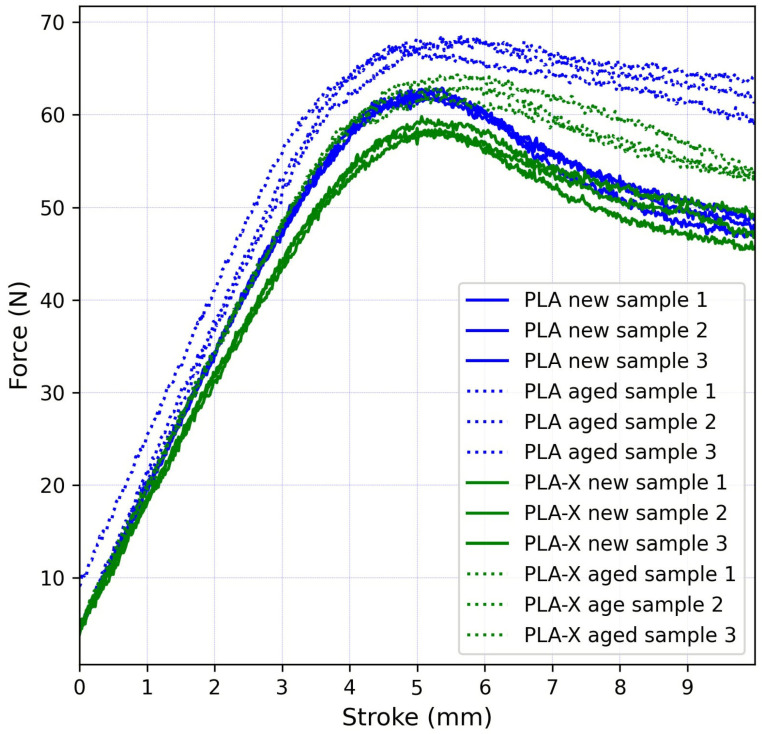
Force–stroke diagram for all samples.

Maximal force values for all samples occurred for a vertical displacement of approximately 5 mm, as can be seen in Figure 3.

When conducting material testing, statistical analysis plays an important role. A commonly used statistical tool in material testing is the *t*-test. The *t*-test allows researchers to compare the means of two groups and determine if there is a significant difference between them. In this study, the *t*-test showed that there was a statistically significant difference between the groups in all four scenarios: the *p*-value for the aged and new PLA samples was 0.0033, for the aged and new PLA-X samples was 0.0121, for the new PLA and PLA-X samples was 0.0136, and for the aged PLA and PLA-X samples was 0.0150.

The traditional approach for analyzing the mechanical properties of a biomaterial using a three-point bending test has the disadvantage that it provides only global information about the material’s behavior. This means that it does not provide detailed insight into the local strain distribution within the material. However, the DIC method in biomedicine offers additional insight, especially in terms of strain localization [34]. By analyzing the strain of the material’s surface using DIC, researchers can obtain detailed information on the strain distribution at different points within the material [35]. Two-dimensional DIC is a non-contact full-field measurement technology widely used in material testing. This method tracks the movement of the stochastic pattern applied to the sample surface during loading. This provides a more comprehensive understanding of the behavior of the material and can be particularly useful for studying materials with inhomogeneous mechanical properties [36].

**Figure 4:**
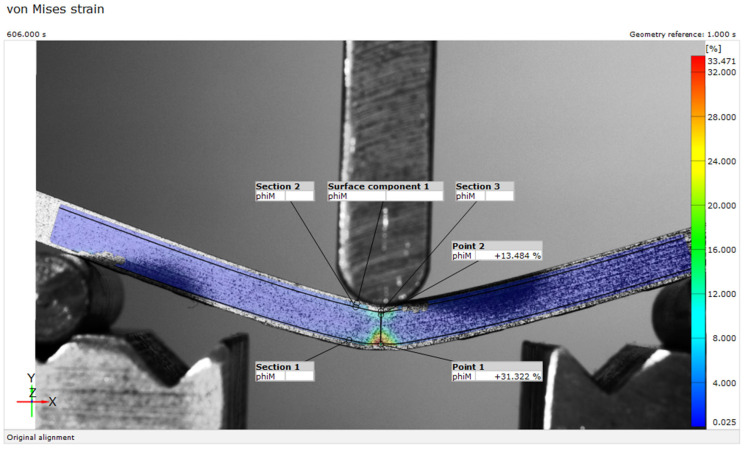
Von Mises strain field for new PLA sample 1 after 10 min of loading.

**Figure 5:**
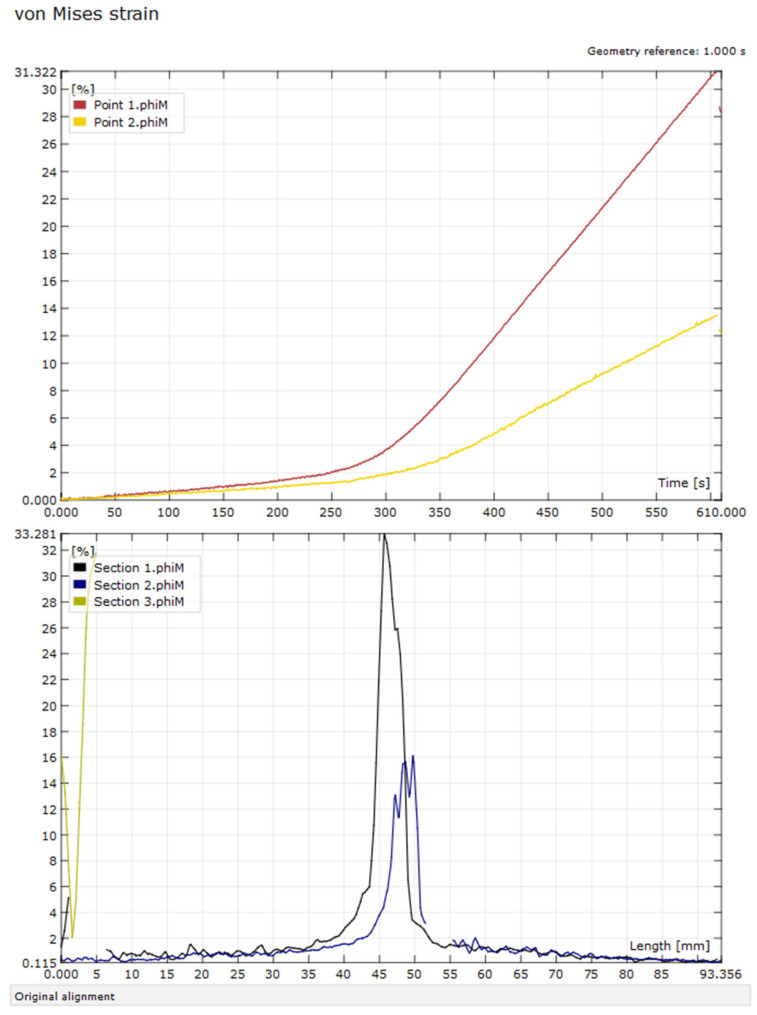
Von Mises strain values for Points 1 and 2 over time (upper diagram) and for Section 1, Section 2 and Section 3 over section length for new PLA sample 1 after 10 min of loading.

The results of the von Mises strain values measured using the 2D-DIC method are presented in this paper. The strain field was analyzed using three sections (Section 1, Section 2 and Section 3) and two points (Points 1 and 2), as shown in Figure 4. Section 1 and Section 2 were horizontal, positioned 1 mm from the lower and upper edge of the sample, respectively. Section 3 was vertical, positioned directly below the loading point. Points 1 and 2 were located at the intersection of Section 3 and Section 1 and Section 2, respectively. Figure 4 shows a representative image of the von Mises strain field results of a new PLA sample after 10 min of loading. The experimental data are also represented in diagrams as time and section length functions, as shown in Figure 5.

The highest strain values were recorded directly below the loading point, in the region of the central 20 mm of the sample (Figure 4). The left and right sides of the sample had strain values below 2%, while the central part of the sample had substantial differences in the lower and upper part of the sample, with values ranging from 33% to 16%, respectively. The strain localization was better visualized in the diagram of Section 1, Section 2 and Section 3, pinpointing the area with critical values (Figure 5). However, more than double the difference was not observed in the traditional approach for analyzing mechanical properties of materials. As the highest measured values were in the central area of the sample, below the loading point, Points 1 and 2 were used for the in-depth comparison of the four material groups.

**Figure 6:**
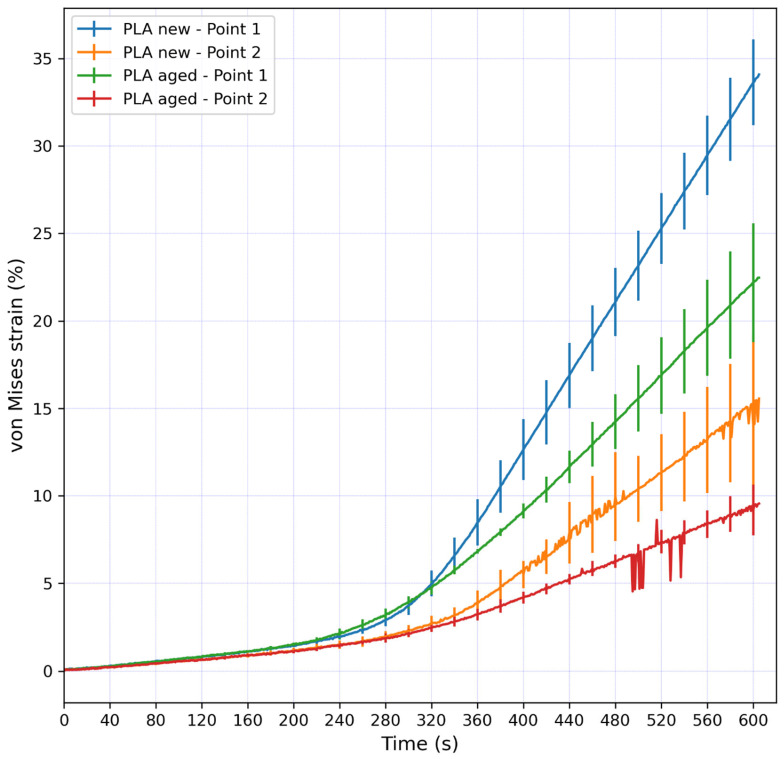
Average von Mises strain values at Points 1 and 2 for new and aged PLA samples. The error bars represent the standard deviation of the measurements for three samples of the same material during loading.

Figure 6 shows the average von Mises strain values at Points 1 and 2 for the new and aged PLA samples. Every line had error bars; the error bars were presented at every twentieth data point, as there were many data points. All the samples showed almost identical trends and strain values in the initial loading phase. At Point 1, the strain values started to differ after approximately 320 s after the start of the experiment, and the difference was constantly increasing until the end of the experiment, reaching the maximal average strain values of around 34% and 23% for the new and aged samples, respectively. At Point 2, the strain values started to differ after approximately 300 s after the start of the experiment, and the difference was constantly increasing until the end of the experiment, reaching the maximal average strain values of around 15% and 9% for the new and aged samples, respectively. The new PLA samples showed higher strain values at both Points 1 and 2 after 300 s of the experiment (300 s corresponded to a 5 mm stroke where the highest force values occurred). At the 5 mm stroke, the strain difference between the new and aged samples were negligible at both Points 1 and 2.

**Figure 7:**
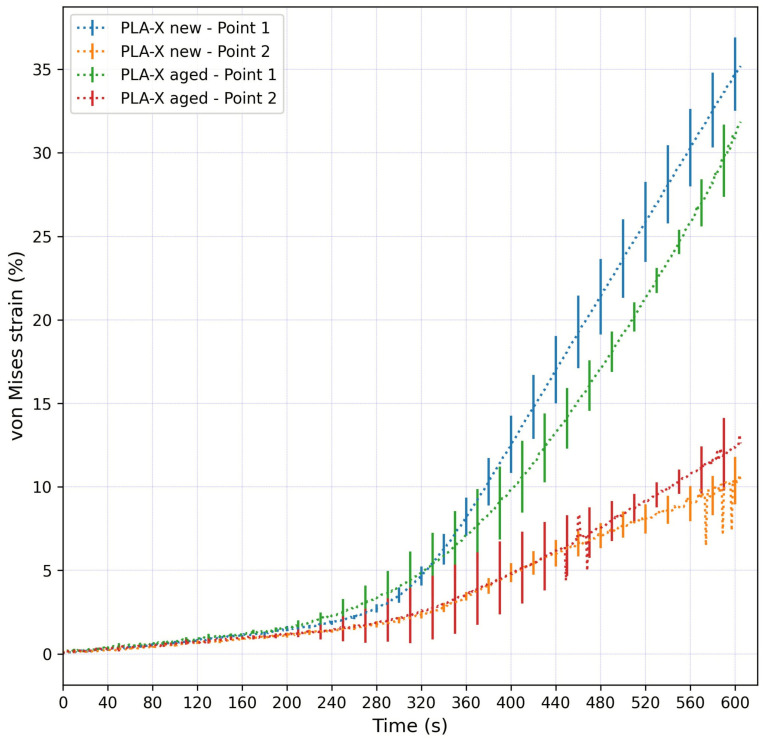
Average von Mises strain values at Points 1 and 2 for new and aged PLA-X samples. The error bars represent the standard deviation of the measurements for three samples of the same material during loading.

Figure 7 shows the average von Mises strain values at Points 1 and 2 for the new and aged PLA-X samples. Every line had error bars; the error bars were presented at every twentieth data point, as there were many data points. All the samples showed almost identical trends and strain values in the initial loading phase. At Point 1, the strain values started to differ after approximately 320 s after the start of the experiment, and the difference was constantly increasing until the end of the experiment, reaching the maximal average strain values of around 35% and 32% for the new and aged samples, respectively. In the period 200–320 s, the aged PLA-X sample showed higher strain values. At Point 2, the strain values started to differ after approximately 450 s after the start of the experiment, and the difference was constantly increasing until the end of the experiment, reaching the maximal average strain values of around 10% and 13% for the new and aged samples, respectively. At the 5 mm stroke, the strain difference between the new and aged samples was negligible at both Points 1 and 2; however, the aged PLA-X sample at Point 1 showed higher strain values for about 1% more than the new sample.

**Figure 8:**
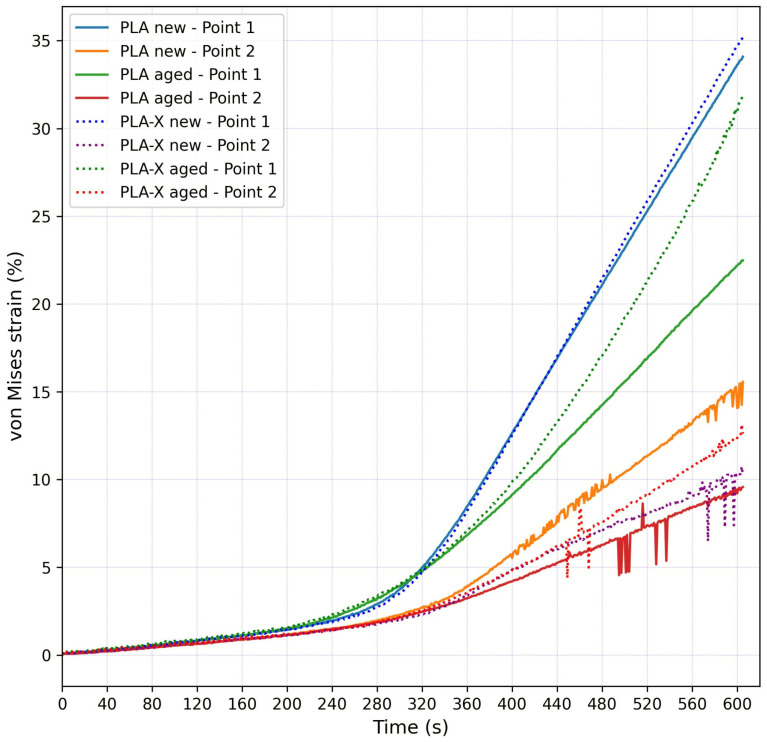
Average von Mises strain values at Points 1 and 2 for new and aged PLA and PLA-X samples.

Figure 8 shows the average von Mises strain values at Points 1 and 2 for the new and aged samples grouped for different materials (the PLA samples were represented with full lines and the PLA-X samples with dotted lines). All the samples for both materials showed almost identical trends and strain values in the initial loading phase. At Point 1, the strain values between the materials started to differ after approximately 320 s after the start of the experiment. The new PLA and PLA-X samples at Point 1 had identical trends, with a difference of 1% at the highest strain values. On the other hand, the aged PLA and PLA-X samples at Point 1 had a significant strain value difference of around 10%, where the PLA-X samples endured much higher strain values. At Point 2, the situation differed. The strain values started to differ after approximately 350 s after the start of the experiment, and the difference increased constantly until the end of the experiment. Unlike for Point 1, the aged PLA-X sample at Point 2 had higher strain values than the new one.

Aging can have a significant influence on the mechanical properties of 3D-printed samples, particularly when it comes to tensile properties and the three-point bending test. Hasan et al. [18] investigated the impact of aging and heat treatment on the tensile properties of PLA printed parts and found that aging had a detrimental effect on the tensile strength and elongation at break of the printed parts. The aging process caused a decrease in both the tensile strength and the elongation at break, indicating a reduction in the overall mechanical performance of the printed samples. On the other hand, the three-point bending test can provide insight into the flexural strength of printed samples. Similar to the work of Hasan et al. [18], the present study showed that aging influences the mechanical properties of 3D-printed materials, in this case the flexural strength. However, limited research is available that directly addresses the influence of aging on the flexural properties of 3D-printed samples using the three-point bending test. Therefore, more research is needed to explore the specific effects of aging on the flexural strength of 3D-printed samples. Nonetheless, it is important to consider the combined results of the tensile and flexural testing to comprehensively evaluate the mechanical properties of the 3D-printed samples and understand their behavior under different loading conditions.

The three-point bending test is a widely used method to evaluate the mechanical behavior of materials under both tensile and compressive stresses. In this test, the sample is subjected to bending forces, leading to tensile stresses on one side and compressive stresses on the other. Points 1 and 2 are positioned in the tension and compression zone of the sample, respectively. The occurrence of tensile and compressive strain and stress in the sample during three-point bending has been extensively studied in various materials (e.g., human bone, carbon fiber-reinforced polymers, dental restorative materials, etc.) and in various contexts (e.g., evaluation of cancellous bone, shape memory alloy beams, etc.). All studies have highlighted the importance of understanding the tension–compression asymmetry on the bending response of materials. The tension–compression asymmetry in 3D-printed samples subjected to three-point bending for biomedical applications is a critical aspect that has garnered attention in materials science and biomedical engineering. The mechanical behavior of 3D-printed materials, particularly in the context of tension–compression asymmetry, has been a subject of interest due to its implications for the design and performance of biomedical devices and implants.

The 2D-DIC method in biomedicine offers benefits such as full-field strain measurement and the ability to extract useful data from traditional characterization tests. It provides a means for developing more accurate material models and can be used for the strain testing of polymeric materials. However, 2D DIC has limitations, such as being restricted to surface measurements and requiring a speckle pattern on the material’s surface for measurement accuracy. The unexpected peak values occurring in the diagrams are the result of the speckle pattern visibility error in the camera images due to lighting changes during the experiment.

## 4. Conclusions

PLA stands out as the predominant material that is extensively applied across a diverse spectrum of uses in both the biomedical and pharmaceutical areas. To finely adjust the PLA properties, a thorough understanding of its essential parameters is crucial. There is already a large amount of data on the FDM process parameters in the literature, especially regarding the use of PLA material. However, the possibilities of utilization of enhanced PLA-X material in the biomedical field have not been studied yet.

The three-point bending test is a valuable method to evaluate the mechanical properties of biomaterials manufactured using 3D printing technology. It allows researchers to assess the strength and fracture behavior of the samples, providing insight into their suitability for various biomedical applications. The use of 3D printing in specimen preparation enables precise control over material composition and microstructure, facilitating the investigation of different printing parameters and advanced materials. Integrating DIC methods into three-point bending tests offers a powerful tool for analyzing the deformation and failure behavior of biosamples, although challenges related to accurate surface tracking must be addressed.

In this study, the statistical analysis of flexural stress (*t*-test) showed that there was a statistically significant difference between the groups in all four scenarios: the aged and new PLA samples, the aged and new PLA-X samples, the new PLA and PLA-X samples, and the aged PLA and PLA-X samples.

The results showed that both the new PLA and the PLA-X samples endured higher strain values at Points 1 and 2 rather than the aged ones, except for the aged PLA-X sample at Point 2, where the new sample had higher strain values. For the first 5 min of the experiment, both materials exhibited identical behavior, after which point significant differences started to occur for both materials, as well as at Points 1 and 2.

The tension–compression asymmetry in 3D-printed samples subjected to three-point bending for biomedical applications is a multifaceted area of research that draws from materials science, nanotechnology, and biomedical engineering. Understanding the mechanical behavior of 3D-printed materials under tension and compression is crucial for optimizing the performance of biomedical devices, implants, and materials used in various biomedical applications.

The DIC method enhances the analysis of mechanical properties by providing localized strain information that is not available through the traditional three-point bending test alone.

This research focused solely on the laboratory mechanical testing of PLA and PLA-X materials, without additional biocompatibility and biodegradability tests. Further research will include the testing of 3D-printed PLA and PLA-X dental grafts.

## Figures and Tables

**Table 1:** Maximal force and flexural stress for all specimens.

Material	Sample Number	New Sample		Aged Sample	
		Maximal Force, N	Flexural Stress, MPa	Maximal Force, N	Flexural Stress, MPa
**PLA**	1	62.82	56.53	67.73	60.95
	2	62.45	56.20	68.87	61.98
	3	63.31	56.98	68.55	61.70
	Average Value	**62.86**	**56.57**	**68.38**	**61.55**
	St. dev.	0.4307	0.3165	0.5907	0.4341
PLA-X	1	58.70	52.83	64.67	58.21
	2	59.99	53.99	63.69	57.32
	3	58.83	52.94	62.77	56.49
	Average Value	**59.17**	**53.25**	**63.72**	**57.34**
	St. dev.	0.7094	0.5213	0.9538	0.7009

## Data Availability

Data can be requested from the corresponding author.
